# Cross-Domain Priming From Mathematics to Relative-Clause Attachment: A Visual-World Study in French

**DOI:** 10.3389/fpsyg.2018.02056

**Published:** 2018-11-05

**Authors:** Céline Pozniak, Barbara Hemforth, Christoph Scheepers

**Affiliations:** ^1^Laboratoire de Linguistique Formelle, CNRS, Paris Diderot University, Paris, France; ^2^Institute of Neuroscience & Psychology, University of Glasgow, Glasgow, United Kingdom

**Keywords:** priming, arithmetic, psycholinguistics, eyetracking, visual world paradigm, relative clause attachment, language, French

## Abstract

Human language processing must rely on a certain degree of abstraction, as we can produce and understand sentences that we have never produced or heard before. One way to establish syntactic abstraction is by investigating structural priming. Structural priming has been shown to be effective within a cognitive domain, in the present case, the linguistic domain. But does priming also work across different domains? In line with previous experiments, we investigated cross-domain structural priming from mathematical expressions to linguistic structures with respect to relative clause attachment in French (e.g., la fille du professeur qui habitait à Paris/the daughter of the teacher who lived in Paris). Testing priming in French is particularly interesting because it will extend earlier results established for English to a language where the baseline for relative clause attachment preferences is different form English: in English, relative clauses (RCs) tend to be attached to the local noun phrase (low attachment) while in French there is a preference for high attachment of relative clauses to the first noun phrase (NP). Moreover, in contrast to earlier studies, we applied an online-technique (visual world eye-tracking). Our results confirm cross-domain priming from mathematics to linguistic structures in French. Most interestingly, different from less mathematically adept participants, we found that in mathematically skilled participants, the effect emerged very early on (at the beginning of the relative clause in the speech stream) and is also present later (at the end of the relative clause). In line with previous findings, our experiment suggests that mathematics and language share aspects of syntactic structure at a very high-level of abstraction.

## Introduction

Abstract syntactic structures enable us to produce and understand sentences that we have never heard or produced before. Beyond these syntactic representations, human sentence processing requires cognitive resources in varying amounts depending on complexity of the sentences. A question that is still debated is how specific these abstract syntactic structures and the required cognitive capacities are to language processing (Koelsch et al., 2004; Fedorenko et al., 2007; Fedorenko et al., 2009; Scheepers et al., 2011; Amalric and Dehaene, 2016; see also Patel, 2003; Scheepers, 2003; Scheepers and Sturt, 2014; Van de Cavey and Hartsuiker, 2016). To answer this, two main classes of questions for resource sharing between cognitive domains have been discussed in the literature:

(1) *Sharing of structure building resources:* Are abstract representations and/or procedures in language different from other domains, such as mathematics or music? Or do the domains share representations and/or operations for processing? If there are shared representations and/or procedures, which level of representation do they concern? Do they concern syntactic (structural) representations of linguistic, mathematical, or musical representations or do they concern their meaning, i.e., semantic or conceptual representations?
(2) *Sharing of processing capacity*: Independent of linguistic representations, does the linguistic domain share cognitive resources with other domains like music or mathematics, meaning that domains require the same additional cognitive resources in cases of cognitive load (e.g., to process complex linguistic, mathematical, or musical structures)?

Much of the literature in psycholinguistics and cognitive psychology focused on the second research question (shared cognitive capacity) or some combination of the two questions. The experiment presented in this paper focused on the sharing of structure building resources. It showed that linguistic and mathematical expressions shared abstract structural representations or structure building procedures leading to mathematical priming effects on sentence processing. Priming studies within (Bock, 1986; Pickering and Branigan, 1998) or even across languages (Hartsuiker et al., 2004) have been proposed to find out more about the nature of linguistic abstractions. In particular, crosslinguistic priming effects are considered a major argument for shared representations between the first and second language of a bilingual speaker. We applied a very similar approach to cross-domain sharing of structure building resources. Before presenting our experiments, we discuss diverging approaches to the status of linguistic representations in relation to other cognitive domains.

A very strict separation between the linguistic domain and others claiming the absence of any shared resources has been suggested by Amalric and Dehaene (2016) for the relationship between language and mathematics. In their study, they aimed to find out whether mathematical capacities are active in similar brain areas as language competence (for instance, the language semantics network) or rather in separate brain circuits. They ran fMRI experiments in which they scanned the brains of mathematicians and non-mathematicians while they had to evaluate the truth of meaningful and meaningless mathematical and non-mathematical statements such as (1a,b) and (2a,b).

[1] a. A finite left-invariant measure over a compact group is bi-invariant.
  b. In finite measure, the series expansion of the roots of a holomorphic map is reflexive.

[2] a. In ancient Greece, a citizen who could not pay his debts was made a slave.
  b. The Greek mythology is the smallest alcohol derived from the VAT.

The results showed that left-hemispheric brain regions generally associated with language competence were not activated for meaningful mathematical statements in professional mathematicians. Conversely, regions associated with mathematical capacities were not activated during meaningful non-mathematical statements. Amalric and Dehaene (2016) concluded from their studies that high-level mathematical thinking and language do not activate the same regions in the brain and thus do not draw on the same resources. However, when looking at their experimental design, the authors’ conclusions appear to concern mainly the representation and storage of mathematical knowledge compared to general knowledge. Thus, with respect to the two research questions proposed above, this study was mostly related to possible shared relations on the semantic level and possibly with respect to general cognitive resources. The experiments did not concern possible overlap with respect to the syntactic processing of mathematical and linguistic expressions. This means, high-level mathematical knowledge seemed to activate brain regions typically involved with space and number and not the semantic network which is typically activated by encyclopedic knowledge. However, these facts do not rule out that some of the rules and processes for the two domains are shared at the syntactic level. The experiment presented in this paper is mainly about this latter aspect.

In favor of shared structure building resources, Patel (2003) suggested the *shared syntactic resource hypothesis*. This hypothesis implies that domains like music and language demand syntactic rules for processing (linguistic or musical elements need rules to integrate them into larger units such as sentences or musical phrases). Even though these rules themselves are probably specific to their domains, they might employ shared basic structure building resources, e.g., common operations to be executed in the same neural areas. An example for this may be operations to reactivate preceding elements in order to integrate new ones. With respect to shared cognitive capacities, Kljajević, 2010 suggested that some domains like language and music share the same syntactic working memory resources (see also Fedorenko et al., 2007, 2009). However, sharing some cognitive resources for processing does not imply shared structure building resources, i.e., that a connection can be established via abstract representations across domains (and thus, priming across domains; see Koelsch et al., 2004). Domains could just refer to the same domain general cognitive resources to process complex structures (shared capacities). Cross-domain priming studies allow for a more direct test of the connection between the linguistic domain and non-linguistic domains with respect to abstract representations.

Some of the clearest evidence established so far can be found in Scheepers et al. (2011) (see also Scheepers, 2003; Scheepers and Sturt, 2014; Van de Cavey and Hartsuiker, 2016), who found a connection between the linguistic domain and mathematics by looking at relative clause attachment *(e.g., he met the daughter of the teacher who lived in Paris)* in English. Before presenting our experiment, we describe these findings, and the rationale behind them, in more detail.

## Previous Evidence

Scheepers et al. (2011) studied in how far mathematical expressions influence relative clause attachment. Relative clause attachment has the interesting property that it presents a syntactic ambiguity with a close correspondence in certain mathematical expressions (as explained further below). Taking an example from Spanish, in sentences like [3], the relative clause can either attach “high” to the first noun phrase (*la criada*) or “low” to the second noun phrase (*la actriz*). Interestingly, different languages have different basic attachment preferences: the relative clause in French (or Spanish, Portuguese, German) will more often attach to the first noun phrase (high attachment), whereas the relative clause in other languages like in English will more frequently attach to the second noun phrase (low attachment).

[3] Algúien disparó contra la criada de la actriz [que estava en el balcón].
  Someone shot the maid of the actress [that was standing on the balcony]. (Cuetos and Mitchell, 1988).

Attachment preferences for relative clauses can be quite different across and within languages depending on the particular construction, showing that they can be influenced by a variety of factors: the anaphoric status of the relative pronoun (Hemforth et al., 2000), length and information structure (Hemforth et al., 2015), prosody (Fodor, 2002), syntactic (Gibson et al., 1996) or pragmatic properties (Gilboy et al., 1995), or presence or absence of pseudo-relatives in the grammar of the respective language (Grillo and Costa, 2014).

In this paper, we aimed to find out whether these preferences can be changed via structural priming, particularly via priming from non-linguistic structures. The hierarchical structure ambiguity involved in relative clause attachment, i.e., integrating some structural element locally (low-attachment) or non-locally (high-attachment) is not specific to linguistic processing and can be found in other domains like mathematics, music, and possibly in a variety of other cognitive domains (see also Van de Cavey and Hartsuiker, 2016). Therefore, relative clause attachment constitutes a key phenomenon to study whether different domains like mathematics and linguistics have shared representations and whether a connection between the domains can be found in terms of cross-domain structural priming.

**FIGURE 1:**
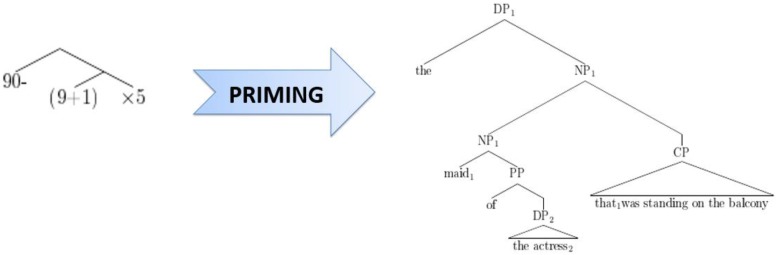
Correspondence between 90–(9 + 1)^∗^5 and high attachment of a relative clause.

**FIGURE 2:**
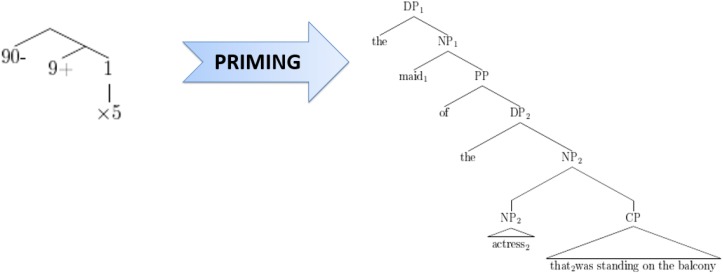
Correspondence between 90–(9 + 1)^∗^5 and low attachment of a relative clause.

Scheepers et al. (2011) noticed a certain resemblance between the structural alternatives for sentences like [3] on the one hand and mathematical expressions like 90-(9 + 1)^∗^5 versus 90-9 + 1^∗^5 on the other hand. Apart from giving different results, these two mathematical expressions have hierarchical structures comparable to, respectively, high versus low attachment of a relative clause in a sentence like [3], and can actually be represented in similar ways. For example, in Figure 1, the expression 90-(9 + 1)^∗^5 [where the final multiplication operator takes scope over a complex expression (9 + 1) on its left] is analogous to high attachment of a relative clause (where the relative clause, or “CP,” takes scope over the entire preceding complex noun phrase), while in Figure 2, the expression 90-9 + 1^∗^5 (where the final operator takes scope over the most recent number on the left) corresponds to low attachment of a relative clause (where the CP takes scope over the most recent noun phrase, or “DP,” on its left).

Figures 1, 2 illustrate that at an abstract level, the hierarchical structure of mathematical expressions can be analogous to that of linguistic expressions. However, there is an important difference between mathematical expressions and relative clause sentences with respect to their ambiguity. Indeed, when considering mathematical operator-precedence rules, mathematical expressions like 90-(9 + 1)^∗^5 and 90-9 + 1^∗^5 are not ambiguous at all. By contrast, sentences with relative clause attachment like *Someone shot the maid of the actress that was standing on the balcony* remain ambiguous on the surface. The main research question of Scheepers et al. (2011) was whether unambiguous mathematical expressions (like the ones discussed above) influence relative clause attachment via structural priming, which would suggest shared (abstract) structural representations between mathematics and language, or shared procedures to process them, i.e., shared structure building resources.

To answer this question, they set up off-line sentence completion experiments in English. Participants were presented with a questionnaire composed of equations to solve and sentence preambles to complete (starting with the relative pronoun, see Table 1). Critical relative clause items were preceded by mathematical expressions to solve (equations analogous to the high-attachment or low-attachment option for relative clause sentences, and “control” equations that did not entail hierarchical structuring).

**Table 1:** Example of an item in the experiment from Scheepers et al. (2011).

Category	Item
High attachment equation	90 - (5 + 15)/15
Low attachment equation	90 - 5 + 15/15
Control equation	5 + 15
Incomplete sentence to fill	The tourist guide mentioned the bells of the church that…


Their results showed a priming effect with more high attachment completions after “high” attaching mathematical expressions and more low attachment completions after “low” attaching mathematical expressions, but interestingly, only in subgroups of participants who were adept in solving the mathematical equations correctly (i.e., business and math students). Another subsample of participants (psychology students) did not show reliable cross-domain structural priming effects, presumably due to a lack of knowledge of the arithmetic operator-precedence rules, as suggested by a high number of mathematical errors in that group (see also Scheepers and Sturt, 2014).

To address this problem, Scheepers et al. (2011) ran another experiment where they added redundant brackets to the critical mathematical equations [e.g., 90-((5 + 15)/15) or 90-5 + (15/15), respectively]. This time, psychology students made far fewer mathematical mistakes and showed clear cross-domain structural priming effects.

Thus, the experiments from Scheepers et al. (2011) (see also Scheepers and Sturt, 2014; Van de Cavey and Hartsuiker, 2016) suggest that linguistic structural processing (more specifically, sentence completion) can be influenced by structural processing in a non-linguistic domain, in this case the mathematical domain.

## The Present Study

While being fairly conclusive in terms of shared structural representations across different cognitive domains, the previous research leaves many questions unanswered. The one we are interested in is whether this cross-structural priming effect can also be generalized to on-line language comprehension, which is less prone to metalinguistic or strategic effects.

To address this question, we conducted an experiment using an on-line comprehension paradigm, namely *visual-world eye-tracking* (e.g., Cooper, 1974; Tanenhaus et al., 1995). This technique combines spoken language with a simultaneously presented visual scene, measuring how auditory language comprehension affects scene perception (more specifically, attention-allocation to syntactic interpretation-relevant referents in the scene) in real time. We chose this paradigm because it has previously been successfully used to investigate language-internal structural priming (from reading to listening) of constituent order in German (Scheepers and Crocker, 2004), or of priming of ditransitive structures in English, for example (Arai et al., 2007).

Another question addressed by our study is whether cross-structural priming from mathematical equations to relative-clause attachment can also be observed in French where relative clause attachment preferences differ from those in English. As discussed earlier, English exhibits a general low-attachment preference for relative clauses, whereas French (the language used in the present study) shows a general high-attachment preference. Indeed, for the notion of cross-domain structural priming to bear substance, it is important to demonstrate that it is independent from language-specific structural biases.

For the current experiment, we expected early as well as late effects of priming from mathematical expressions to relative clause attachment, particularly for participants with good mathematical knowledge and less so for mathematically less adept participants. Similar to Patel (2003), we assumed that basic processing operations for building hierarchical structures are shared between mathematical and linguistic expressions. Thus, participants with good knowledge of the relevant mathematical operations should show fixations corresponding to a stronger tendency to attach the relative clause high after “high attachment” equations than after “low attachment” equations at the beginning of the relative clause, and also (potentially) toward the end of the sentence. No clear predictions could be made at this point for participants with low mathematical knowledge.

## Experiment

Scheepers et al. (2011) found an influence from mathematical expressions on relative clause attachment in English. Still, they (and others) only focused on language production and used off-line questionnaires, which only provided indirect access to linguistic processing. Our study extended this work by setting up an on-line paradigm (visual-world eye-tracking) in another language, i.e., French, a high-attachment preference language. Moreover, we studied priming in comprehension where priming effects have been shown somewhat less consistently (see Traxler et al., 2014, for a discussion).

An important fact about French is that it has *different types* of relative clauses depending on the verb in the main clause, and this peculiarity (which does not exist in English) can have a modulating influence on relative clause attachment preferences (Grillo and Costa, 2014). Therefore, it was imperative to pre-test our French materials in order to confirm the general high attachment preference for relative clauses.

### Norming Study

According to Grillo and Costa (2014), relative clauses introduced by a perceptual verb are called *pseudo-relatives*, which are structurally different from traditional relative clauses because they refer to events and will modify the verb and not any of the nouns. As an illustration, the relative clause in [5a] only denotes a property of the lawyer while in [4a] it can also denote an event. It is also possible to pronominalize the head noun of a pseudo-relative clause like in [4b] contrary to [5b]. In that case, [4] has two readings: a pseudo-relative reading and a traditional relative reading, and according to Grillo and Costa (2014), the pseudo-relative reading is preferred, meaning that the relative clause takes the first noun as its subject (here, the son), an interpretation that superficially resembles high attachment (and this relative clause attaches to the verb). This type of relative clause exists in French but not in English.

[4] a. Le médecin voit le fils de l’avocat qui court.
  The doctor sees the son of the lawyer that runs.
  b. Le médecin le voit qui court.
  The doctor sees him that runs.

[5] a. Le médecin déteste le fils de l’avocat qui court.
  The doctor hates the son of the lawyer that runs.
  b. ^∗^Le médecin le déteste qui court.
  ^∗^The doctor hates him that runs.

Thus, in order to avoid having different structures of relatives which could add noise to our experimental results, we decided to have the same main clause for every item: *Voici …*. Indeed, Grillo and Costa (2014) attribute the high attachment preference for relative clauses in French mainly to the existence of pseudo-relative clauses. By excluding pseudo-relative readings in French, the high attachment preference for sentences like [5] is therefore less certain^1^. This is why we decided to run a forced choice task with our items to test attachment preferences.

#### Participants

Fifty native speakers of French participated in the experiment (mean age: 35 years old, σ = 17). They were recruited via the RISC^2^ platform. Three participants were excluded because their first language was not French.

#### Materials

The items consisted of 30 sentences containing an ambiguous relative clause, followed by two possible interpretations of the relative clause (see [6]). The interpretations were either about the first noun (NP1, [6a]) or the second noun (NP2, [6b]).

[6] Voici le cuisinier de l’ingénieur qui va finir ce sur quoi il travaillait.
  Here we have the cook of the engineer who will finish what he was working on.
  a. Le cuisinier va finir ce sur quoi il travaillait.
  The cook finished what he was working on.
  b. L’ingénieur va finir ce sur quoi il travaillait.
  The engineer finished what he was working on.

Orders of interpretation-paraphrases (NP1-related first or NP2-related first) were counterbalanced across the 30 items per presentation list. Two lists were created with order of paraphrases swapped across lists. Thirty filler sentences of an independent experiment were added to the lists. The order of presentation was randomized individually for each participant.

#### Procedure

The experiment was run online via the Internet-based platform *IbexFarm* (Drummond, 2010). For each trial, participants read a sentence with an ambiguous relative clause and they had to choose which of the two interpretations was more natural and acceptable. The experiment lasted about 20 min.

#### Results

No significant difference was found concerning the order of the interpretation paraphrases. Participants chose NP1-attachment paraphrases 71% of the time and NP2-attachment paraphrases 29% of the time. A logistic regression model with simple intercept showed a significant difference between NP1-and NP2-attachment: β = 1.44, *z* = 4.85, *p* < 0.001. Thus, we found that the NP1-modifying high-attachment interpretation was strongly preferred, contrasting with English which is more biased toward low attachment.

### Eye-Tracking Experiment

We ran an Eye-Tracking experiment using the Visual World paradigm in French. We manipulated mathematical equations in order to investigate directly their priming effect on ambiguous relative clause comprehension.

#### Participants

Thirty six native speakers of French participated in the eyetracking experiment, all living in Paris at the time of the experiment (mean age: 30 years old, σ = 11). All participants gave written informed consent before taking part and the study was approved by the College of Science and Engineering Committee at the University of Glasgow (Application Number: 300150090) as well as by the Comiteì d’Ethique pour les Recherches en Santeì at the University Paris Sorbonne Cité (CERES; Application Number: 2018-34).

Participants were divided into two groups^3^ (more information in the procedure section): a *mathematical knowledge* group and a *no mathematical knowledge* group, see also Scheepers and Sturt (2014).

#### Materials

**FIGURE 3:**
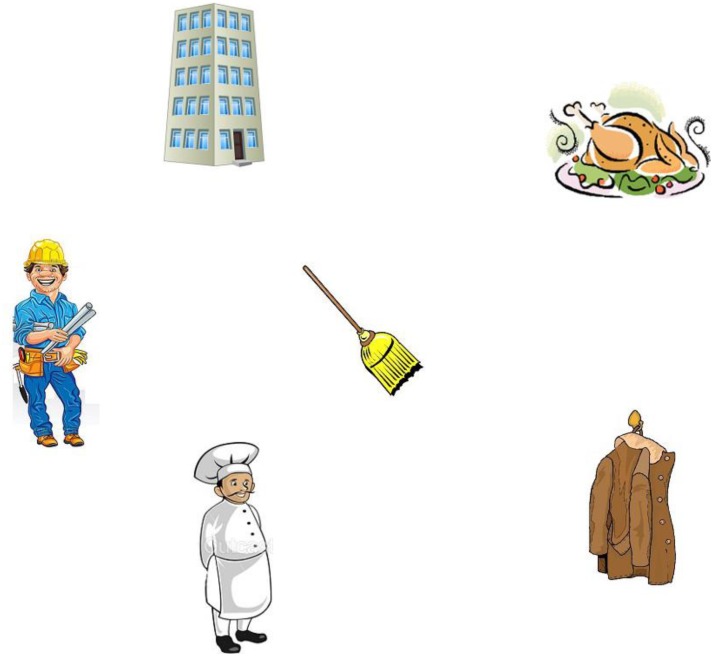
Example of a picture used as target visual-world materials.

For this experiment, we used the SR Research Experiment Builder 2.1.140 [Computer software] (2017) for the setup. Thirty experimental items (see Appendix) were constructed, each comprising two types of prime equations (see below), plus a picture and a spoken sentence as target visual-world materials. Each of the 30 target pictures (Figure 3) comprised cartoon-like depictions of several objects and characters in an arbitrary layout (with changing positions across items): two human protagonists (e.g., a chef and an engineer, serving as referents for NP1 and NP2 in the spoken sentences, respectively), two objects associated with those protagonists (e.g., a roast chicken and a tall building), and two unrelated distractor objects (e.g., a broom and a coat). The association between nouns and their related objects was based on semantic and pragmatic associations as estimated by the authors of this paper^4^.

Audio stimuli were spoken French sentences like [6], which ended in an attachment-ambiguous relative clause (e.g., English translation: “*Here we have the cook of the engineer who will finish what he was working on*”). Relative clauses were constructed to be “semantically neutral,” i.e., they were constructed to be equally plausible modifiers of NP1 or NP2. The sentences were spoken by a female native French speaker using neutral intonation and digitized for later presentation using Audacity and checked in PRAAT (Boersma and Weenink, 2018).

Each item included two types of priming equations – high-attachment (e.g., 4 + (6-2)/2) and low-attachment (e.g., 4 + 6-2/2) – which were paired with one of the pictures (composed of four objects and two human characters) plus a spoken sentence as target for visual-world trials. See Table 2 for example equations in the two conditions (high attachment and low attachment). The equations were structurally equivalent to the high- and low-attachment equations used in Scheepers et al. (2011). They were easily solvable without using a calculator and always resulted in a non-negative whole number. In high-attachment equations, a multiplication or division operation at the end was preceded by a bracketed term (either an addition or a subtraction) on its left, whereas in low-attachment equations, the brackets were omitted so that the final multiplication or division took scope over the most recently encountered number. Participants were thus presented with 30 experimental items, 15 items per condition.

**Table 2:** Example equations in the two conditions.

Conditions	Equations
High attachment	77-(14 + 21)/7
	56-(5 + 3)^∗^4
Low attachment	77-14 + 21/7
	56-5 + 3^∗^4


**FIGURE 4:**
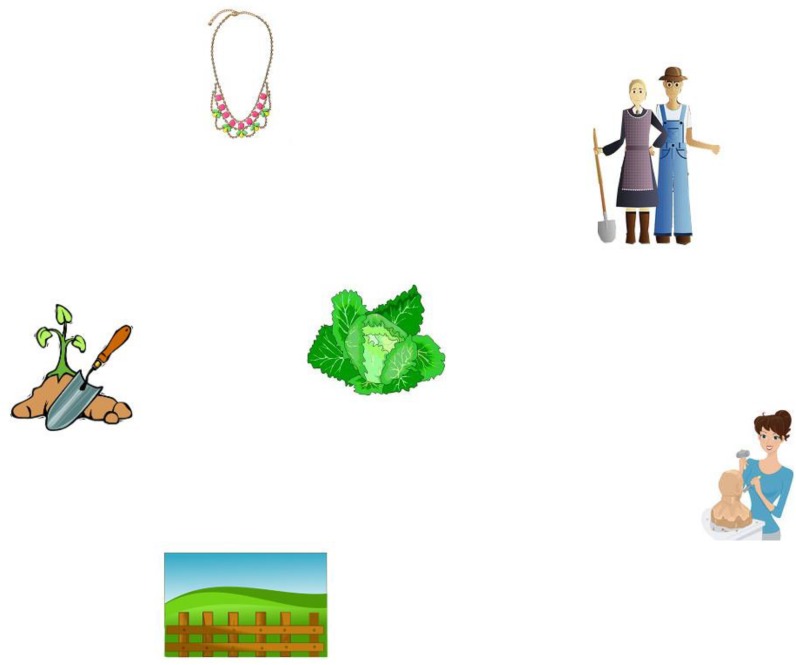
Example of a picture used as filler visual-world materials.

Fifty six fillers were also added. These comprised 26 equations (structurally different from the critical prime equations, Table 3) and 30 pictures combined with auditory French sentences that ended in unambiguous relative clauses (e.g., English translation: “*Here we have the gardeners who will offer a necklace to the sculptor*,” Figure 4)^5^.

**Table 3:** Example equations for the fillers.

Fillers
10-5 + 22
(17 + 11)/7
(27-(8 + 1))/9


The two experimental conditions (high attachment equation/low attachment equation) were presented to participants following a Latin square design: Two counterbalanced presentation lists were generated such that (a) half of the experimental picture-sentence combinations (serving as targets) were preceded by high-attachment prime equations and the other half by low-attachment prime equations and (b) item-condition combinations were swapped across the two lists. As our dependent variable, we looked at fixations on the NP1 object vs. the NP2 object, meaning fixations on the NP1 object out of fixations on both objects.

The materials per list were pseudo-randomized, ensuring that each experimental pair of prime equation and picture-sentence combination was separated from the others by at least one filler trial, randomly chosen from the pool of filler equations and filler visual-world trials. Because of the latter, there was no regular sequencing of mathematical versus visual-world trials (i.e., it was not the case that a mathematical trial was always followed by a visual-world trial or vice versa). To ensure that participants paid attention to the pictures and sentences, comprehension questions were included after 16 of the (filler or experimental) visual-world trials. Eight concerned the spoken sentence (e.g., “was a cowboy mentioned?”), and eight referred to the picture itself (e.g., “was there a broom in the picture?”).

The incorrect answer option for the mathematical equations always corresponded to the result of linear processing of the equation without taking operator-precedence rules into account (i.e., the result according to an incorrect structuring of the equation). Positioning of correct/incorrect answer options was counterbalanced across items.

#### Hypothesis

Based on our experimental design, upon hearing “*qui va finir ce sur quoi il travaillait*” (“*who will finish what he was working on*”) in the sentence, we expected participants to look more at the NP1-related object (roast chicken) in Figure 3 if they had been primed toward assuming a high-attachment structure, and to look more at the NP2-related object (building) if they had been primed toward assuming a low-attachment structure. In principle, such priming effects could manifest themselves as early as during encountering the relative pronoun (“qui”) in the sentence if participants expect references to NP1- and NP2-related objects in the relative clauses. Alternatively, such effects could take more time to emerge (e.g., toward the end of the relative clause, when all interpretation-relevant information is available). We expected that mathematical priming would affect attachment preferences particularly for mathematically adept participants, who solved equations correctly while no clear predictions were made for mathematically less adept participants.

#### Procedure

Before the experiment started, participants had to solve the following equation: 1 + 2^∗^3 = ? Depending on the answer (7 or 9), they were either in the mathematical knowledge group (7) or the no mathematical knowledge group (9).

The eye-tracking experiment was run with an SR Research Eyelink 2 system in a sound attenuated booth. We recorded eye fixations from the dominant eye based on the Test Miles (1930). Each experiment started with a nine-point calibration.

**FIGURE 5:**
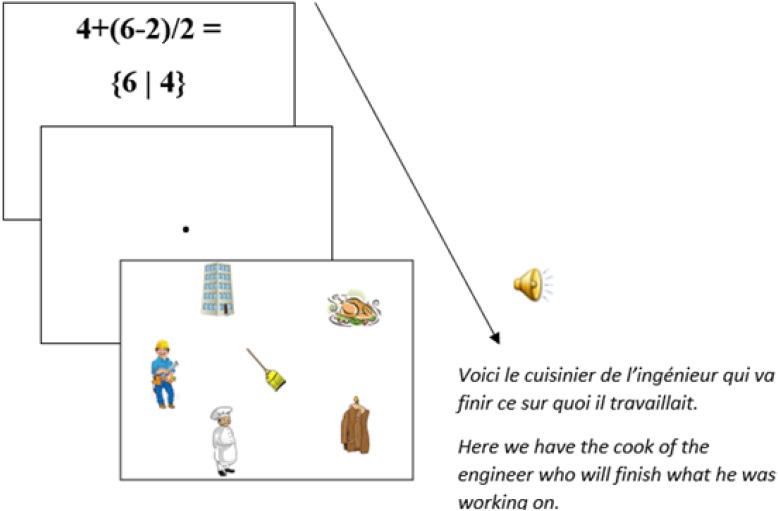
Presentation sequence for a critical prime (equation) – target (visual-world trial) pair of stimuli.

Participants were instructed that there were two types of trials: (i) mathematical trials and (ii) picture-sentence trials^6^. Each picture-sentence trial was preceded by the presentation of a fixation dot for drift-correction. On mathematical trials, participants saw an equation on the screen with two possible answer options below it (see Figure 5). They had to choose the correct answer by pressing the left button on a gamepad for the answer on the left, and the right button for the answer on the right.

**FIGURE 6:**
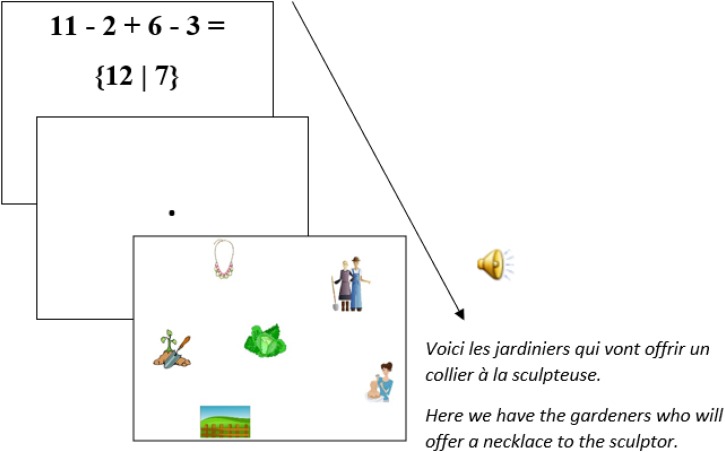
Presentation sequence for a non-critical prime (equation) – target (visual-world trial) pair of stimuli.

In picture-sentence trials, they had to look at pictures while listening to related spoken sentences. Participants were informed that after some of the picture-sentence trials, a question related to either the picture or the sentence would appear, which they were to answer with either the right-hand button (for “yes”) or the left-hand button (for “no”) on the gamepad. Eye movements were recorded throughout the presentation of each picture, which stayed on screen for 5000 ms. The speech recordings for the sentences started playing right after the onset of each picture presentation. Figure 5 illustrates the presentation sequence for a critical prime-target pair of trials and Figure 6 for a non-critical prime-target pair of trials. There was a practice session before the actual experiment (four trials) to familiarize participants with the task.

Importantly, participants remained unaware about the pairing of critical prime-target trials until debriefing at the end of the experimental session.

#### Analysis Methods

We used the R package EyetrackingR (Dink and Ferguson, 2015) to analyze the Eye-Tracking data. We then analyzed them by running growth curve analyses (Mirman et al., 2008). Independent variables (predictors) were Prime Condition (high attachment vs. low attachment), Time and Group (mathematical knowledge and no mathematical knowledge). Dependent variables (outcome variables) were fixations on the NP1-related object or on the NP2-related object. For the results presented in this paper, we focused on the NP1- and NP2-related objects because they were the relevant objects for the interpretation of the ambiguous relative clause, rather than the protagonists. Fixations on NP1 and NP2 can be found in supplementary materials^7^). Random variables were participants and items.

We chose growth curves due to the fact that eye fixations on the target objects should change depending on what part of the sentence participants are listening to at a certain moment. Thus, time is a very strong predictor that needs to be taken into account. Growth-curve analyses included time as a continuous predictor with the dependent variable being categorical (binary).

Growth-curves for the visual world data were analyzed with Bayesian generalized linear mixed models (Sorensen et al., 2016, among others) using the R package Rstan. Our main motivation behind choosing a Bayesian modeling framework was that previous frequentist analyses (using the lme4 package) often failed to converge when a maximal random effects structure justified by the design was used (cf. Barr et al., 2013). Bayesian models are much less prone to convergence failure, even with relatively small data sets. Another advantage of Bayesian analysis is that it directly tests the likelihood of the hypothesis of interest, contrasting with indirect null-hypothesis testing as in frequentist frameworks. Moreover, we express the uncertainty of the effects by the means of credible intervals.

Our hypothesis was that a priming effect would show up especially for the mathematical knowledge group. In other words, looks to the NP1-related object would be more frequent in the high attachment condition for the mathematical knowledge group. The opposite pattern was expected for the NP2-targeted object.

Fixed effects were Time, Group, and Prime Condition as well as their interactions. Random effects were participants and items, and we also included random slopes for the effects of Prime Condition and Group for items, as well as their interactions. For participants, we only included random slopes for Prime Condition.

As for the coding, the group factor was coded 1 for the mathematical knowledge group and -1 for the no mathematical knowledge group. The Prime Condition factor was coded 1 for high attachment and -1 for low attachment. Normal distributions with μ = 0, SD = 10 on a logit scale were used as weakly informative priors.

To determine the shape of the fixed effect of time, we ran three linear mixed models with simple intercepts to test which polynomial function corresponds best to our data (see Appendix C)^8^. Then, we ran Bayesian models with 4 chains and 6000 iterations each. Model convergence was verified graphically and by inspecting model coefficients in R.

According to our hypothesis, potential priming effects may show up either at the beginning of the relative clause or toward the end of the sentence (when all the information is available). We therefore analyzed the data per participant group in two separate time-windows: (i) 200 ms before the beginning of the relative clause until 200 ms after the beginning of the relative clause, and (ii) 500 ms before the end of the sentence until the end of the sentence.

Concerning the pre-test (1 + 2^∗^3 = ?), 20 participants were initially attributed to the *mathematical knowledge* group and 16 participants to the *no mathematical group*. However, after inspecting the results more closely, we found that the accuracy rate for the condition equations within the experiment was lower than 70% for two participants from the *mathematical knowledge* group. We decided to put them in the *no mathematical knowledge* group, leading to two balanced groups with 18 participants each. We present the analysis with this change in the results section^9^.

#### Results

##### Comprehension accuracy

To be sure that participants were engaged to the task, we included comprehension questions on the pictures (*Did you see an umbrella on the picture?* / *Was the little girl sick?*). Participants from the *mathematical knowledge* group answered 87% of the comprehension questions correctly, while participants from the *no mathematical knowledge* group answered 85% of the comprehension questions correctly. This difference was not significant.

##### Mathematical performance

When looking at the percentage of correct answers to the equations, we found that participants from the mathematical knowledge group answered 84% of the high-attachment equations and 97% of the low-attachment equations correctly. By contrast, participants in the no mathematical knowledge group answered 12% high-attachment equations and 19% low-attachment equations correctly. As for the structurally simpler filler equations, participants in the mathematical knowledge group answered them correctly 96% of the time, and participants of the no mathematical knowledge group 90% of the time.

These results suggest that participants in the mathematical knowledge group generally “understood” the structure of the prime equations, whereas participants in the no mathematical knowledge group had difficulty particularly with identifying the correct operator-precedence relations in the equations (as already suggested by the initial classification).

##### Eye fixations on the NP1 (vs. NP2)-related object

As explained before, we focus on fixations on the NP1- and the NP2-object in the results section. Fixations to other objects can be found in supplementary materials^10^. Probabilities of looks in this section are looks to the NP1-object out of NP1- and NP2-object fixations. We only show looks to the NP1-related object because looks to the NP2-object would be their mirror image.

**FIGURE 7:**
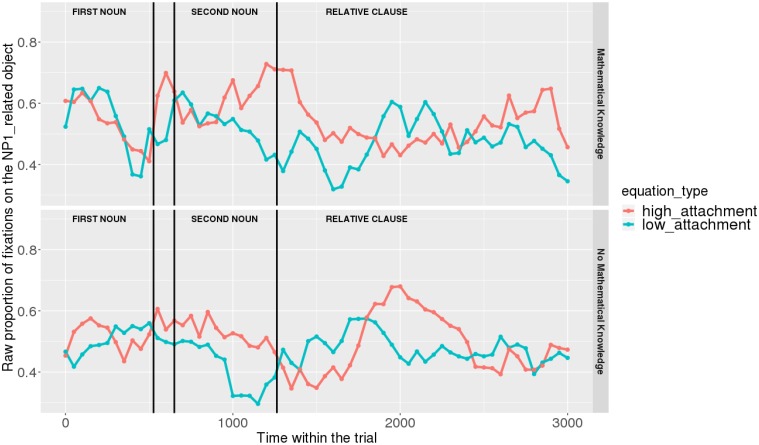
Raw proportion of fixations on the NP1-related object (roasted chicken) in relation to Prime Condition for both groups (Participants variable, t1).

**FIGURE 8:**
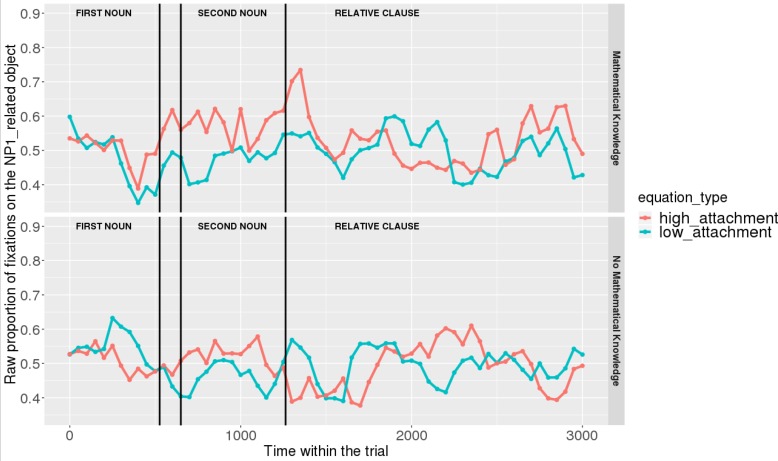
Raw proportion of fixations on the NP1-related object (roasted chicken) in relation to Prime Condition for both groups (Items variable, t2).

Figures 7, 8 show raw proportions of looks to the NP1-related object (out of fixations to NP1-related object + fixations to NP2-related object) in relation to Prime Condition (high attachment vs. low attachment) every 50 ms, from the first noun to the end of the sentence (3023 ms). Results from the *mathematical knowledge* group (upper part of the Figure), and from the *no mathematical knowledge* group (lower part of the Figure) are presented. The blue line refers to fixations to the NP1-related object under the low attachment condition while the red line concerns fixations to the NP1-related object under the high attachment condition.

**FIGURE 9:**
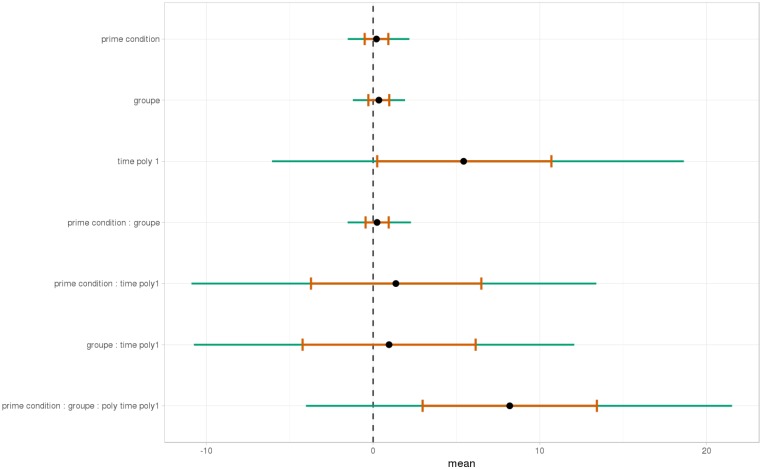
Posterior distribution of independent variables for the first part: during the beginning of the relative clause (1063–1463 ms).

**FIGURE 10:**
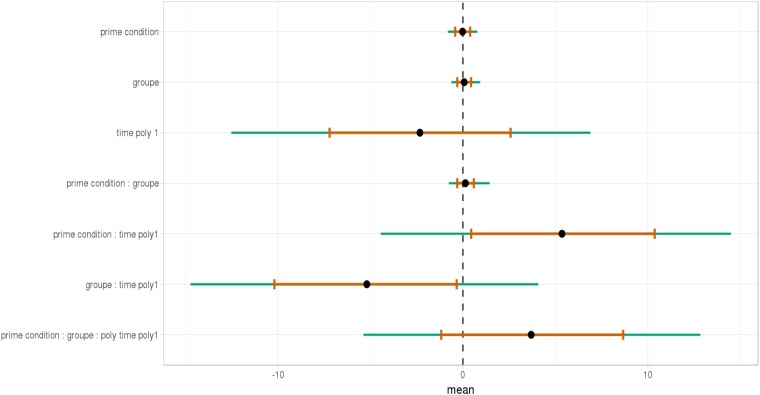
Posterior distribution of independent variables for the second part: from the relative to the end of the sentence (2523–3023 ms).

As shown, respectively, in Figures 9, 10, an interaction of Time, Group, and Prime Condition was observed around the beginning of the relative clause, more precisely in the region 200 ms before and 200 ms after the beginning of the relative clause (poly1, 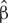 = 8.20, 95% CrI = [1.95, 14.34], P(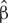) < 0 < 0.006) and (less so) 500 ms before the end of the sentence until the end of the sentence (poly1, 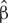 = 3.69, 95% CrI=[-1.17 8.67], P(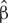) < 0 <0.07). This means that there was an effect of mathematical attachment over time and that the difference between high attachment and low attachment differs across mathematical knowledge groups over time.

When sub-setting the data by group, we found different patterns for the *mathematical knowledge* group and the no *mathematical knowledge* group in the two time-windows.

**FIGURE 11:**
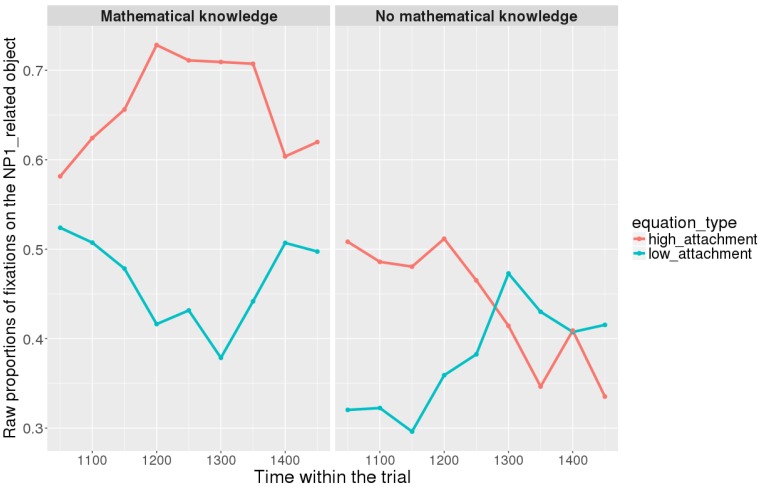
Proportions of looks toward the NP1-related object in each of the two groups at the beginning of the relative clause (1063–1463 ms). Red curves: high-attachment prime condition; blue curves: low-attachment prime condition (Participants variable).

**FIGURE 12:**
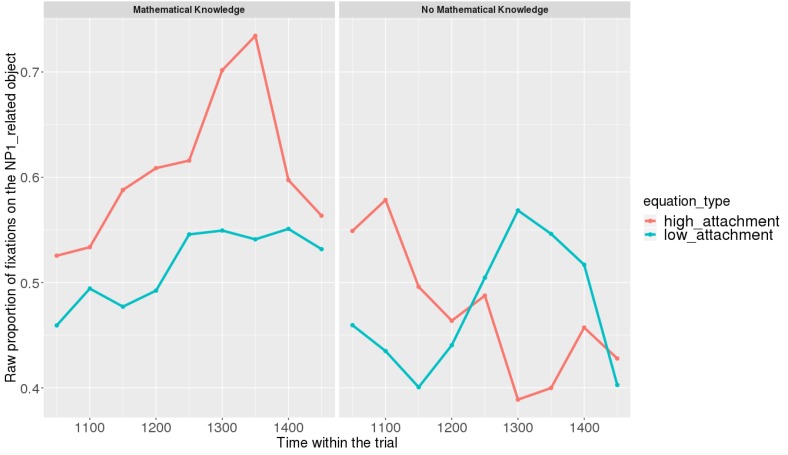
Proportions of looks toward the NP1-related object in each of the two groups at the beginning of the relative clause (1063–1463 ms). Red curves: high-attachment prime condition; blue curves: low-attachment prime condition (Items variable).

**FIGURE 13:**
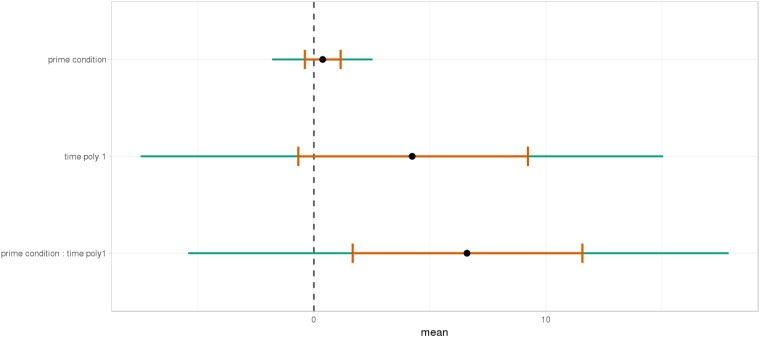
Posterior distribution of independent variables for the first part (1062–1462 ms) in the *mathematical knowledge* group.

**FIGURE 14:**
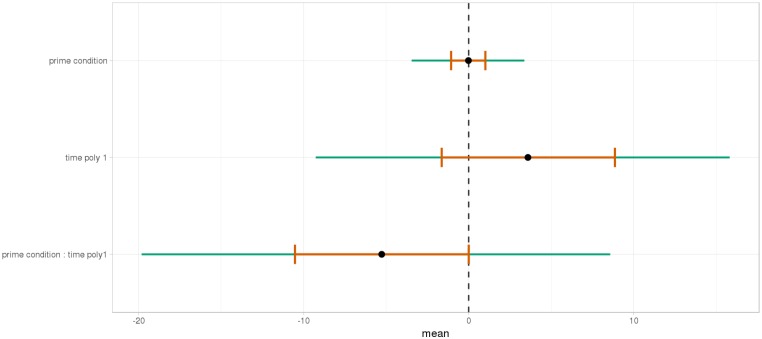
Posterior distribution of independent variables for the first part (1064–1464 ms) in the no *mathematical knowledge* group.

At the beginning of the relative clause in Figures 11, 12, we found an interaction between Prime Condition and Time (Figure 13, poly1: 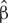 = 6.43, 95% CrI = [0.58, 12.36], *P*(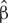) < 0 < 0.02)^11^ for the mathematical knowledge group. We also found an interaction for the no mathematical knowledge group but with a different pattern over time: the initial difference between high attachment and low attachment disappears over time (see the results for this group on the right in Figure 10), which explains why there is an inverse pattern for the interaction between time and Prime Condition in Figure 14 for this group (poly 1: 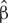 = 6.43, 95% CrI = [-11.54, 1.03, *P*(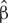) < 0 = 0.95.

**FIGURE 15:**
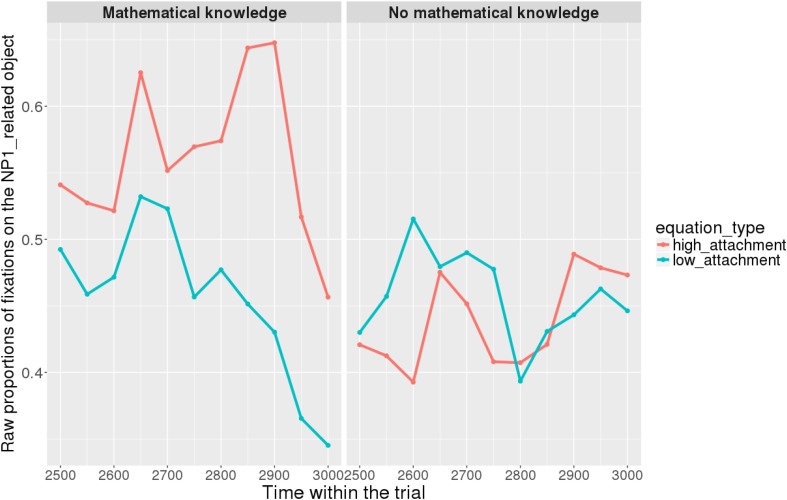
Proportions of looks toward the NP1-related object in each of the two groups at the end of the sentence (2523–3023 ms). Red curves: high-attachment prime condition; blue curves: low-attachment prime condition (Participants variable).

**FIGURE 16:**
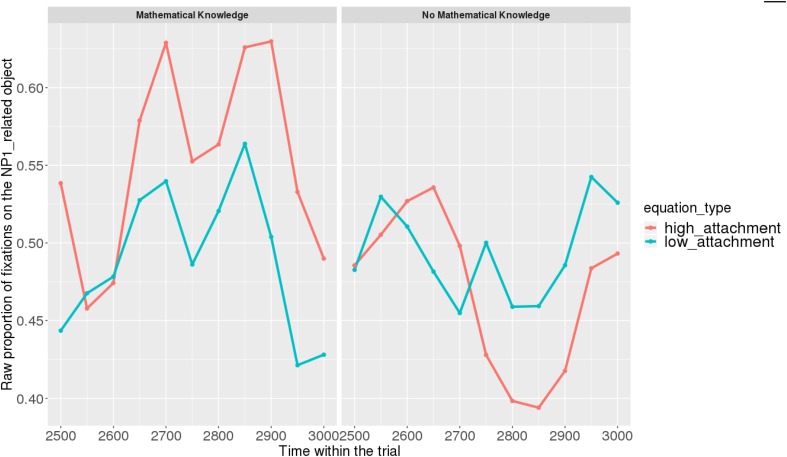
Proportions of looks toward the NP1-related object in each of the two groups at the end of the sentence (2523–3023 ms). Red curves: high-attachment prime condition; blue curves: low-attachment prime condition (Items variable).

**FIGURE 17:**
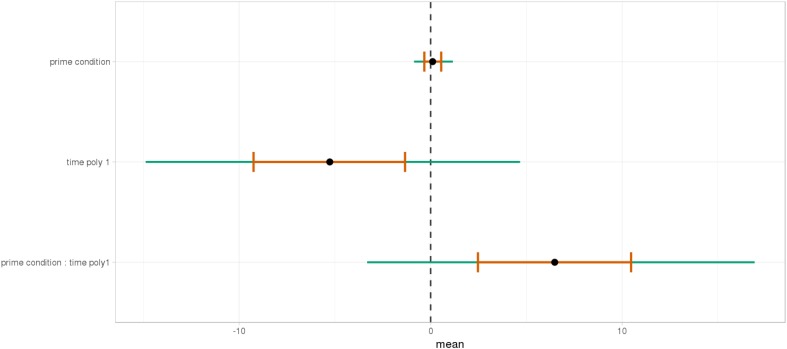
Posterior distribution of independent variables for the second part (2515–3015 ms) in the *mathematical knowledge* group.

**FIGURE 18:**
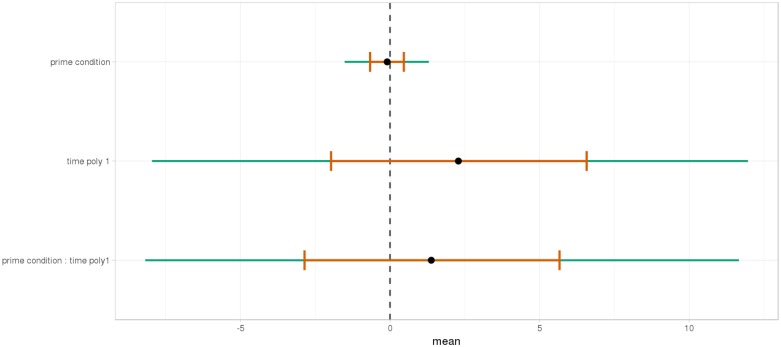
Posterior distribution of independent variables for the second part (2530–3030 ms) in the no *mathematical knowledge* group.

At the end of the sentence (Figures 15, 16), the interaction of time and Prime Condition is still there for the *mathematical knowledge* group (Figure 17 poly 1: 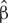 = 6.91, 95% CrI=[1.99, 11.82], *P*(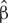) < 0< 0.003 but not for the no *mathematical knowledge* group (Figure 18, poly 1: 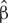 = 1.42, 95% CrI=[-3.79, 6.60], *P*(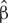) < 0< 0.30).

Overall, it can be concluded that cross-domain structural priming effects emerged very early on (even before the onset of the relative pronoun itself) and were especially robust and persistent in the *mathematical knowledge* group of participants compared to the no *mathematical knowledge* group.

## General Discussion

The present study replicates and extends aspects of earlier off-line results by Scheepers et al. (2011) and others (e.g., Scheepers and Sturt, 2014; Van de Cavey and Hartsuiker, 2016) in interesting and important ways. Specifically, we investigated structural priming across cognitive domains – from mathematical equations to relative-clause attachments in spoken sentences – in French, a language known to contrast with English in terms of general relative clause attachment preferences: whereas English speakers/listeners usually prefer to attach relative clauses low to the most recent NP in a sentence, French speakers typically prefer high attachment of relative clauses, as was also confirmed in the pre-test of our materials. More importantly, the present study is the first to investigate cross-domain structural priming effects within the context of *on-line* sentence comprehension: indeed, the visual-world eye-tracking paradigm allowed us to pin down more precisely than previous studies at which point during processing of an attachment-ambiguous relative clause potential cross-domain syntactic priming effects would emerge. Another potentially important aspect of the present study is that the visual world paradigm is arguably less prone to strategic effects, as it requires no metalinguistic judgment or explicit syntactic attachment decisions by the participants (i.e., they just “look and listen” and are unlikely to become aware of their syntactically driven visual biases). It may be argued that participants leave interpretations underspecified when no explicit interpretation is required as Swets et al. (2008) have suggested for relative clause attachment ambiguities in reading. This may, however, be different for ambiguity resolution in the visual world paradigm where interpretations are done in a reduced universe. Recent studies on pronoun resolution using the “look and listen” paradigm showed that participants’ fixations reflect their interpretational preferences about as much as when questions about the interpretation are asked (Colonna et al., 2015).

Given that structural priming relies on the accurate processing of the structural primes (mathematical equations in this case), clear structural priming effects were in fact only expected for mathematically adept participants – in line with Scheepers et al. (2011) who found that without additional help in solving the mathematical primes (such as additional redundant brackets in the prime equations) mathematically less skilled participants would not only struggle to solve the priming equations correctly, but also show a generally less robust priming effect from mathematical equations to linguistic expressions. The latter was also evident in the *no mathematical knowledge* group of participants in our study: although there was evidence of cross-domain structural priming in this group as well, effects were less pronounced and more distributed over time than in the *mathematical knowledge* group who were much more likely to solve the prime equations correctly, and who showed cross-domain structural priming effects during the earliest stages of integrating the ambiguous relative clause into the preceding sentence context (specifically, around the onset of the relative pronoun in the spoken target sentence).

The early effect is interesting in terms of when exactly during sentence processing cross-domain structural priming effects would emerge. Scheepers et al. (2011) actually speculated about potential processing mechanisms that could underlie their off-line results. In what they called the *incremental-procedural account* they proposed that, just as the processing of linguistic materials, processing of mathematical equations structured like A + (B + C)^∗^D, respectively, A + B + C^∗^D would proceed in an incremental “left-to-right” fashion. This would trigger a form of mild (monotonic) revision as soon as the final multiplication or division operator is encountered at the end of the equation, because the operator-precedence rules require that this operation is applied before the preceding term [(B + C), respectively, C] is added; in “high-attachment” equations like A + (B + C)^∗^D, the final operator would combine with a complex expression on its left (B + C), whereas in “low-attachment” equations like A + B + C^∗^D it would combine with the most recent number (C) on its left. In analogy to this, a form of mild revision is also likely to take place when encountering a relative pronoun after incrementally processing a partial sentence such as *The tourist guide mentioned the bells of the church…* (*that*) because (i) the sentence is obviously not complete at this point and (ii) the relative pronoun needs to be integrated with a preceding noun phrase. In analogy to the mathematical primes, this relative pronoun can either combine with a complex noun phrase on its left (e.g., *the bells of the church*, yielding high attachment) or with the most recent noun phrase on its left (e.g., *the church*, yielding low attachment). Scheepers et al. (2011) proposed that it is this combinatorial choice that is affected by cross-domain structural priming, and the present findings (particularly for the mathematically adept group of participants) seem to support this *incremental-procedural* view: Evidence for structural priming became manifest as soon as the relative pronoun (*qui*) was encountered in the speech stream, i.e., in a time window from 200 ms before to 200 ms after the onset of the relative pronoun. This early onset of effects is in line with the hypothesis that cross-domain structural priming influences the immediate (incremental) syntactic integration of relative pronouns during sentence processing. However, one probably needs to be cautious with this interpretation, as a different treatment of the response proportions in a supplemental analysis (using NP1-related object fixations in proportion to fixations on all the other objects available^12^) suggested more delayed effect onsets relative to the relative pronoun, while still showing clear support for cross-domain structural priming.

What are the more general implications of cross-domain structural priming between mathematics and language processing? In the introduction, we discussed seemingly conflicting evidence suggesting a strict separation between language and mathematics in the brain (e.g., Amalric and Dehaene, 2016). We would argue that this conclusion is too narrowly focused on the semantics of linguistic vs. mathematical expressions and does not apply to the processing of abstract hierarchical structure as investigated here and in previous studies. It is also important to keep in mind that shared processing resources for mathematical and linguistic expressions must not necessarily rely on networks that are *specialized* for linguistic and/or mathematical processing. Indeed, it is conceivable that potentially shared representations may be processed at some separate, *domain-general* level that is independently accessible by brain networks that are more specialized for mathematics and language. Such a view also concurs with findings from Varley et al. (2005) showing that patients with severe agrammatic aphasia can nevertheless perform well at various mathematical tasks.

To conclude, in line with previous findings (e.g., Scheepers et al., 2011), the present visual-world eye-tracking experiment suggests that mathematics and language share aspects of syntactic structure at a very high level of abstraction. These effects appear to be largely independent of baseline RC-attachment preferences (low attachment in English, high attachment in French). More importantly, for mathematically skilled participants at least, the present study appears to indicate that cross-domain priming from mathematics to language influences the very earliest stages of integrating a relative clause into the prior sentence context, in line with an *incremental-procedural* account of hierarchical structure priming.

## Author Contributions

CP and CS conceived the idea and designed the experiments. CP and BH designed the pretest. CP analyzed the data. CP, BH, and CS interpreted the results and revised the content. CP wrote the initial draft.

## Conflict of Interest Statement

The authors declare that the research was conducted in the absence of any commercial or financial relationships that could be construed as a potential conflict of interest.

## Footnotes

1 Still, it can be argued that the *voici* construction resembles a pseudo-relative construction (Lahousse and Karssenberg, 2016). Nevertheless, since the construction was systematically the same for all items, the preference should be the same, and it cannot explain differences in attachment preferences due to priming.

2 http://www.risc.cnrs.fr

3 Participants were unaware of this classification, and none of them produced a result other than 9 or 7 (except one participant, whose data was thus excluded from analysis).

4 Although we did not run a norming study to confirm the association between the nouns and their related objects as judged by the authors of this paper, looks at the targeted object during the ambiguous relative clause to be discussed in the results section showed the associations were relevant for the task.

5 See https://www.dropbox.com/sh/qq7ujz55dgfzgtp/AAAOsJK4aXmjRY1o_mo3J_-Wa?dl==0 for more details (Appendix A).

6 The instructions can be found in Appendix B https://www.dropbox.com/sh/qq7ujz55dgfzgtp/AAAOsJK4aXmjRY1o_mo3J_-Wa?dl==0.

7 Available at www.dropbox.com/sh/qq7ujz55dgfzgtp/AAAOsJK4aXmjRY1o_m o3J_-Wa?dl==0.

8 This is available on https://www.dropbox.com/sh/qq7ujz55dgfzgtp/AAAOsJK4aXmjRY1o_mo3J_-Wa?dl==0.

9 The former analysis is available in Appendix D on https://www.dropbox.com/sh/qq7ujz55dgfzgtp/AAAOsJK4aXmjRY1o_mo3J_-Wa?dl==0.

10 Available at https://www.dropbox.com/sh/qq7ujz55dgfzgtp/AAAOsJK4aXmjRY1o_mo3J_-Wa?dl==0.

11 The fact that *P*() < 0 < <0.02 means that the probability of the hypothesis “<0” is very low, meaning that participants from the *mathematical knowledge* group look more to NP1-targeted object across the relevant time period when the Prime Condition is high attachment. This is not the case for the participants from the *no mathematical knowledge* group with *P*() < 0 = 0.95, where the priming effect disappears over time.

12 This is available on https://www.dropbox.com/sh/qq7ujz55dgfzgtp/AAAOsJK4aXmjRY1o_mo3J_-Wa?dl==0.

## APPENDIX

### Appendix: Materials Used for the Spoken Sentences and the Equations (Items)

#### Spoken Sentences

**Table:**

Number	Spoken sentence	Translation
Item1	Voici le fils du jeune papa qui veut faire ce qu’il préfère.	Here we have the son of the father who fancies riding what he likes best.
Item2	Voici le plombier du technicien qui va réparer ce qu’on lui a demandé.	Here we have the plumber of the technician who will set up what he was asked to.
Item3	Voici le père du petit garçon qui s’apprête à avoir sa boisson.	Here we have the father of the baby boy who is about to have his drink.
Item4	Voici le cuisinier du touriste qui va préparer ce qu’il a besoin de faire.	Here we have the cook of the tourist who will prepare what he needs to.
Item5	Voici le jardinier du cuisinier qui va couper ce qu’il doit faire.	Here we have the gardener of the cook who will cut what he has to.
Item6	Voici le tailleur de l’architecte qui s’apprête à créer un chef d’ oeuvre.	Here we have the tailor of the architect who is about to create a masterpiece.
Item7	Voici l’architecte du producteur qui va dévoiler sa dernière création.	Here we have the architect of the film producer who will reveal his latest creation.
Item8	Voici la cartomancienne de la femme d’affaires qui va lire ce qu’elle préfère d’habitude.	Here we have the fortune teller of the businesswoman who will read what she’s used to.
Item9	Voici la costumière de l’actrice qui va continuer à travailler sur son dernier projet.	Here we have the costume designer of the actress who will continue working on her latest project.
Item10	Voici le postier du boulanger qui doit livrer ce qu’il possède.	Here we have the postman of the baker who will deliver what he has.
Item11	Voici le sculpteur de la fleuriste qui va présenter sa nouvelle oeuvre.	Here we have the sculptor of the florist who will present his recent work.
Item12	Voici l’électricien du conducteur qui va changer ce qui ne fonctionne plus.	Here we have the electrician of the driver who will change what doesn’t work anymore.
Item13	Voici la fille de la dame qui va boire sa boisson préférée.	Here we have the daughter of the lady who will sip her favorite drink.
Item14	Voici la grand mère de l’adolescente qui va chercher ce qu’elle a l’habitude d’utiliser.	Here we have the grandmother of the teenage girl who will fetch what she’s used to.
Item15	Voici le professeur du serveur qui va au travail.	Here we have the teacher of the waiter who is going to work.
Item16	Voici le photographe de l’homme d’affaires qui va acheter ce dont il a besoin.	Here we have the photographer of the businessman who will purchase what he needs.
Item17	Voici le confiseur du menuisier qui va créer ce qu’il fait de mieux.	Here we have the confectioner of the carpenter who will create what he’s best known for.
Item18	Voici la mère de la jeune fille qui va chercher son objet favori.	Here we have the mother of the girl who will fetch her favorite item.
Item19	Voici l’électricien du professeur qui va travailler sur ce qu’il est censé faire.	Here we have the electrician of the teacher who will work on what he’s supposed to.
Item20	Voici la fille de la dame qui va porter son vêtement préféré.	Here we have the daughter of the lady who will wear her favorite clothes.
Item21	Voici la fillette de la femme de ménage qui va prendre ce qu’elle cherchait.	Here we have the baby girl of the cleaner who will grab what she was looking for.
Item22	Voici le fermier du chanteur qui va devoir vendre son objet préféré.	Here we have the farmer of the pop star who’ll have to sell his favorite possession.
Item23	Voici le grand père du végétarien qui va manger son déjeuner.	Here we have the grandfather of the vegetarian who will eat his lunch.
Item24	Voici le fils du jeune papa qui va prendre son diner.	Here we have the son of the father who will have his dinner.
Item25	Voici l’ami du soldat qui va mettre sa tenue habituelle.	Here we have the friend of the soldier who will put on his usual outfit.
Item26	Voici le cuisinier de l’ingénieur qui va finir ce sur quoi il travaillait.	Here we have the cook of the engineer who will finish what he was working on.
Item27	Voici le fermier du menuisier qui va finir son travail.	Here we have the farmer of the carpenter who will finish what he started to work on.
Item28	Voici la fille du chimiste qui va mettre sa tenue habituelle dans la matinée.	Here we have the daughter of the chemist who will put on her usual outfit in the morning.
Item29	Voici le père du jeune garçon qui va lire ce qu’il préfère.	Here we have the father of the boy who will read what he’s most interested in.
Item30	Voici l’enfant malade de l’homme d’affaires qui va avoir ce qu’il veut désespérément.	Here we have the sick son of the businessman who will have what he’s desperate for.

#### Equations

**Table:**

Number	Equation
Item1 high attachment	4 + (6-2)/2 = {6 | 4}
Item1 low attachment	4 + 6-2/2 = {4 | 9}
Item2 high attachment	60- (9 + 1) ^∗^ 5 = {10 | 250}
Item2 low attachment	60-9 + 1 ^∗^ 5 = {260 | 56}
Item3 high attachment	10 + (7-5) ^∗^ 3 = {16 | 36}
Item3 low attachment	10 + 7-5 ^∗^ 3 = {36 | 2}
Item4 high attachment	60-(24-12)/3 = {56 | 16}
Item4 low attachment	60-(24-12)/3 = {8 | 32}
Item5 high attachment	41-(8 + 3) ^∗^ 3 = {8 | 90}
Item5 low attachment	41-(8 + 3) ^∗^ 3 = {108 | 42}
Item6 high attachment	7 + (28-4) ^∗^ 2 = {55 | 62}
Item6 low attachment	7 + (28-4) ^∗^ 2 = {62 | 27}
Item7 high attachment	20 + (32-6)/2 = {33 | 23}
Item7 low attachment	20 + (32-6)/2 = {23 | 49}
Item8 high attachment	10 + (20 + 10)/5 = {16 | 8}
Item8 low attachment	10 + (20 + 10)/5 = {8 | 32}
Item9 high attachment	56-(5 + 3) ^∗^ 4 = {24 | 192}
Item9 low attachment	56-(5 + 3) ^∗^ 4 = {216 | 63}
Item10 high attachment	16-(12-4)/2 = {12 | 4}
Item10 low attachment	16-(12-4)/2 = {0 | 2}
Item11 high attachment	31 + (8-5) ^∗^ 2 = {37 | 68}
Item11 low attachment	31 + (8-5) ^∗^ 2 = {68 | 29}
Item12 high attachment	2 + (6 + 4) ^∗^ 3 = {32 | 36}
Item12 low attachment	2 + 6 + 4 ^∗^ 3 = {36 | 20}
Item13 high attachment	45-(27-9)/3 = {39 | 9}
Item13 low attachment	45-(27-9)/3 = {3 | 15}
Item14 high attachment	77-(14 + 21)/7 = {72 | 6}
Item14 low attachment	77-(14 + 21)/7 = {12 | 66}
Item15 high attachment	16 + (24-8)/4 = {20 | 8}
Item15 low attachment	16 + (24-8)/4 = {8 | 38}
Item16 high attachment	10 + (6 + 3) ^∗^ 2 = {28 | 38}
Item16 low attachment	10 + (6 + 3) ^∗^ 2 = {38 | 22}
Item17 high attachment	90-(5 + 15)/5 = {14 | 86}
Item17 low attachment	90-(5 + 15)/5 = {88 | 20}
Item18 high attachment	56 + (6 + 6)/2 = {34 | 62}
Item18 low attachment	56 + (6 + 6)/2 = {65 | 34}
Item19 high attachment	48-(9 + 6) ^∗^ 2 = {66 | 18}
Item19 low attachment	48-9 + 6 ^∗^ 2 = {51 | 90}
Item20 high attachment	4 + (22-4)/2 = {11 | 13}
Item20 low attachment	4 + (22-4)/2 = {24 | 11}
Item21 high attachment	45-(10 + 5) ^∗^ 3 = {90 | 0}
Item21 low attachment	45-(10 + 5) ^∗^ 3 = {50 | 120}
Item22 high attachment	90-(50-30)/10 = {7 | 88}
Item22 low attachment	90-(50-30)/10 = {37 | 1}
Item23 high attachment	70-(25 + 5)/5 = {8 | 64}
Item23 low attachment	70-(25 + 5)/5 = {46 | 10}
Item24 high attachment	1 + (26-1) ^∗^ 4 = {104 | 101}
Item24 low attachment	1 + (26-1) ^∗^ 4 = {23 | 104}
Item25 high attachment	13 + (17-10) ^∗^ 3 = {60 | 34}
Item25 low attachment	13 + (17-10) ^∗^ 3 = {0 | 60}
Item26 high attachment	3 + (2 + 1) ^∗^ 4 = {24 | 15}
Item26 low attachment	3 + (2 + 1) ^∗^ 4 = {9 | 24}
Item27 high attachment	21 + (84-14)/7 = {13 | 31}
Item27 low attachment	21 + (84-14)/7 = {103 | 13}
Item28 high attachment	54-(30 + 12)/6 = {2 | 47}
Item28 low attachment	54-(30 + 12)/6 = {26 | 6}
Item29 high attachment	99-(27-18)/9 = {10 | 98}
Item29 low attachment	99-(27-18)/9 = {70 | 6}
Item30 high attachment	40-(4 + 8) ^∗^ 3 = {84 | 4}
Item30 low attachment	40-(4 + 8) ^∗^ 3 = {60 | 132}
