# Corrigendum: Cross-Domain Priming From Mathematics to Relative-Clause Attachment: A Visual-World Study in French

**DOI:** 10.3389/fpsyg.2018.02560

**Published:** 2018-12-12

**Authors:** Céline Pozniak, Barbara Hemforth, Christoph Scheepers

**Affiliations:** ^1^Laboratoire de Linguistique Formelle, CNRS, Paris Diderot University, Paris, France; ^2^Institute of Neuroscience & Psychology, University of Glasgow, Glasgow, United Kingdom

**Keywords:** priming, arithmetic, psycholinguistics, eyetracking, visual world paradigm, relative clause attachment, language, French

In the original article, we neglected to thank the source of visual materials, Eunice Fernandes, Holly Branigan, and Moreno Coco, as well as Olaf Mikkelsen for his help in running the experiments.

A correction has been made to the Acknowledgements:

## Acknowledgments

This work was partly funded by the Laboratoire d'Excellence Empirical Foundations of Linguistics (ANR-10-LABX-0083). We thank Yair Haendler for his helpful advice for the bayesian analysis and Olaf Mikkelsen for his contribution in running the experiments. We also thank Eunice Fernandes, Holly Branigan, and Moreno Coco for kindly letting us use their visual materials for the eye-tracking experiment.

The authors apologize for this error and state that this does not change the scientific conclusions of the article in any way. The original article has been updated.

### Conflict of Interest Statement

The authors declare that the research was conducted in the absence of any commercial or financial relationships that could be construed as a potential conflict of interest.

